# 雷公藤内酯醇与非小细胞肺癌研究进展

**DOI:** 10.3779/j.issn.1009-3419.2013.07.09

**Published:** 2013-07-20

**Affiliations:** 210002 南京，南京大学医学院临床学院，南京军区南京总医院呼吸内科 Department of Respiratory Medicine, Nanjing General Hospital of Nanjing Command, Clinical School of the Medical College of Nanjing University, Nanjing 210002, China

**Keywords:** 雷公藤内酯醇, 肺肿瘤, 肿瘤抑制, Triptolide, Lung neoplasms, Antineoplastic

## Abstract

雷公藤内酯醇对多种非小细胞肺癌（non-small cell lung cancer, NSCLC）细胞系具有杀伤作用，可通过干预细胞周期、激活caspase信号通路、抑制血管内皮生长因子（vascular endothelial growth factor, VEGF）的表达、抑制NF-κB活性等多种途径来促进肺癌细胞死亡。现将雷公藤内酯醇的抑瘤功能及其具体作用机制加以综述，为其在NSCLC中的科学研究及临床应用提供思路。

## Abstract (en)

Triptolide can cure a variety kinds of non-small cell lung cancer cell lines. It can interfere the cell cycle, active the caspase signaling pathway, inhibit the of expression vascular endothelial growth factor (VEGF), inhibit the activation of NF-κB, and through these ways to promote lung cancer cell death. Now the anti-tumor mechanism and effect of triptolide was summarized to provide help for scientific research and clinical applications in non-small cell lung cancer.

肺癌是对人类健康与生命危害最大的恶性肿瘤之一。在我国肺癌已居城市癌症发病率和死亡率的首位。非小细胞肺癌（non-small cell lung cancer, NSCLC）占肺癌的绝大部分，很多患者确诊时即属于肿瘤晚期，不宜手术治疗。接受手术治疗的患者中，术后复发转移率高。而NSCLC对化疗药物不甚敏感，治疗效果有待进一步提高。因此寻找有效的药物治疗NSCLC是医学工作者的科研重心。

雷公藤内酯醇（triptolide, TPL/TL）又称雷公藤甲素、雷公藤内酯，是从卫矛科植物雷公藤中提取的一种含有3个环氧基的二萜内酯化合物，是雷公藤的主要活性成分。它的药理活性强，具有免疫抑制、抗感染、抗生育、抗肿瘤、抗过敏等作用，在体内化学性质稳定，临床上得到了广泛应用，用于治疗类风湿性关节炎、系统性红斑狼疮等各种自身免疫性疾病和器官移植的排斥反应^[1]^。

20世纪70年代Kupchan等^[2]^从台湾产雷公藤中分离出雷公藤内酯醇，并发现其对小鼠白血病细胞株L-1210和P-388都有很强的抑制活性，因此揭开了雷公藤内酯醇抗肿瘤研究的序幕。大量实验研究发现，雷公藤内酯醇可抑制NSCLC细胞增殖、促进凋亡，并可抑制实体瘤的生长。现就近年来雷公藤内酯醇抑制NSCLC的研究进展做一综述。

## 1 TL的生物学特性

TL是具有松香烷骨架结构的二萜类三环氧内酯化合物。外观呈无色针状结晶体，相对分子质量为360，分子式为C_2_OH_24_O_6_，熔点为（mp）226 ℃-230 ℃，不溶于水，易溶于二甲亚砜、丙酮、乙醇等有机溶剂^[3]^。口服或静脉注射后在体内分布以肝脏浓度最高，半衰期约为2 d。其活性基团包括14位碳上羟基、3个环氧基团以及1个五元不饱和内酯环，这些基团是其发挥多种生物活性的结构基础，在一定条件下能与生物大分子中的亲核基团发生反应，从而抑制这些大分子的功能，杀伤肿瘤细胞。

目前通过对TL的进一步提取以及对结构的改造和修饰，已经合成了一系列TL的衍生物，包括雷公藤氯内酯醇（tripchlorolides, T4）、5-羟雷公藤内酯醇[(5R)-5-hydroxytriptolide, LLDT-8]、PG27、PG490-88^[4]^等。

## 2 TL具有抑制NSCLC的活性

宋岚等^[5]^发现TL对体外培养的NSCLC细胞具有明显的抑制作用。TL可抑制A549细胞的生长，并诱导A549细胞的凋亡; 且其作用具有剂量依赖性，随着TL浓度增加，细胞抑制率逐渐增加，凋亡率也逐渐升高。欧阳曙明等^[6]^的研究亦证实TL能明显抑制A549细胞的增殖，并具有时效性和量效性。

罗泳仪等^[7]^将Spc-A1细胞加入不同浓度的TL，发现它对Spc-A1细胞的增殖具有抑制作用，并且抑制率随浓度和时间的增加而增加。TL作用6 h后，随着药物浓度的增加，Spc-A1细胞的体外侵袭能力、趋化性运动能力明显降低，对基质的粘附能力有一定程度降低。另有研究^[8]^证明TL抑制肺癌UISO-LUC-1细胞增殖（ED_50_=10 ng/mL）。其提取物LLDT-8可以抑制A549及NCI-H460细胞的增殖^[9]^。

TL的抑瘤作用在动物实验亦得到证实。Fidler等^[10]^报道TL的水溶性衍生物PG490-88能明显减慢肺癌细胞H23荷瘤裸鼠移植瘤的生长速度，并呈剂量依赖性。0.25 mg/kg的PG490-88即可明显抑制肿瘤的生长，0.5 mg/kg及0.75 mg/kg的PG490-88抑制现象更加明显。高剂量PG490-88可使部分裸鼠移植瘤完全消失。

## 3 TL抑制NSCLC机理

### 3.1 TL干预细胞周期

细胞周期调控的失常在肿瘤发生发展中发挥重要作用。正常细胞周期由G_1_-S-G_2_-M的顺序进程存在多个阻滞点，细胞受到信号调控发生细胞周期阻滞。发生阻滞的细胞一方面可通过修复受损基因、超过阻滞点、继续发育，完成增殖周期; 另一方面能及时启动凋亡系统，清除受损细胞。若阻滞点这一调控功能丧失，则使携带受损基因组的细胞不发生阻滞，从而导致细胞增殖失控，进而最终导致肿瘤的发生发展。雷公藤内酯醇可以通过干预和调节细胞周期发挥作用。研究^[11]^发现低浓度（14 nmol/L）TL使肺腺癌A549细胞阻滞在S期，而高浓度（112 nmol/L）TL则使A549细胞阻滞在G_2_/M期。这个发现是对TL抗肿瘤特性的突破性认识，拓展了对TL抗癌机制的研究，已引起了越来越多的关注，成为肿瘤治疗领域的新热点。

### 3.2 TL激活caspase信号通路

caspase家族是一类半胱氨酸蛋白酶，在调节细胞凋亡过程中起重要作用。caspase启动酶活化后，可激活下游的执行酶。所有的caspase激酶均以无活性的酶原形式存在，一经激活将产生caspase级联反应，最终导致细胞凋亡。caspase-3是细胞凋亡的重要执行者，可降解蛋白激酶、聚合酶、DNA碎裂因子45、Bax等底物，抑制DNA修复并启动DNA的降解，诱导细胞产生凋亡，使细胞不可逆的走向死亡。欧阳曙明等^[6]^发现用TL处理A549细胞后，caspase-3的活性明显升高，推测TL可能通过激活caspase信号通路诱导细胞凋亡。

细胞凋亡的信号传递途径及调控机制复杂。目前认为，细胞凋亡的发生有两条信号传导通路：一条为死亡受体信号通路，细胞膜表面的死亡受体与配体结合，使前体活化，并进一步激活，导致细胞凋亡; 另一条为线粒体信号通路，在多种凋亡诱导因素作用下，线粒体结构和功能破坏，引起细胞色素等促凋亡分子从线粒体释放进入胞浆，通过一系列生物特性，最终引起细胞凋亡。研究^[5]^发现TL处理A549细胞后caspase-3及caspase-9的活性明显升高，这说明线粒体信号通路也参与了TL诱导的细胞凋亡。

### 3.3 TL抑制血管内皮生长因子（vascular endothelial growth factor, VEGF）的表达

VEGF是目前最重要的血管生成因子之一，可诱导内皮细胞增殖和迁移、促进血管生成，在肿瘤的生长与转移中发挥着重要的作用。VEGF与其受体结合后，可释放各种生长因子及细胞因子，增强血管的渗透性，引起血浆蛋白外渗，为肿瘤细胞的生长提供基质，同时刺激血管内皮细胞增殖和迁移，促进新生血管形成，在肿瘤的浸润和转移中起重要作用。有学者发现TL抑制肺癌细胞Spc-A1的侵袭和运动，其作用机制与VEGF的表达下调有关。TL处理Spc-A1细胞后，Spc-A1细胞VEGF mRNA的表达明显减少，Western blot结果显示不同浓度的TL处理24 h后，Spc-A1细胞VEGF蛋白表达明显下调，表达量随药物浓度增加而逐渐下降^[7]^。

### 3.4 TL抑制NF-κB活性

NF-κB是细胞内调节基因转录的核因子，在炎症、应激、细胞生长增殖等过程中参与许多基因的转录调控。NF-κB能促进细胞增殖，抑制细胞凋亡，在肿瘤的发生发展过程中发挥重要作用。文献^[12]^报道TL能通过抑制NF-κB亚单位p65的反式激活作用抑制NF-κB活性来促进A549肿瘤细胞凋亡。

肿瘤坏死因子（tumor necrosis factor, TNF）、肿瘤坏死因子相关凋亡诱导配体（TNF-related apoptosis-inducing ligand, TRAIL）通过死亡受体途径诱导肿瘤细胞凋亡的过程中由于激活了NF-κB，使细胞对凋亡耐受。研究^[13]^证实TL可以增强肿瘤细胞对TNF-α及TRAIL诱导的细胞凋亡敏感性，能有效地抑制TNF-α介导的NF-κB激活，同时抑制TNF-α对细胞凋亡抑制因子（inhibitor of apoptosis protein, IAP）家族c-IAP1和c-IAP2的诱导作用，从而加强TNF-α诱导肿瘤细胞凋亡的作用。

## 4 TL的毒副作用

TL的安全窗口较窄LD_50_/ED_50_=4（LD_50_：半数致死量，ED_50_：半数有效量），临床用药安全隐患大^[14]^。TL可以使肾小管上皮细胞变性、坏死，严重时可能发生急性肾功能衰竭而死亡; 对肠胃影响较大，会出现恶心、呕吐、腹痛、腹泻、转氨酶升高等现象，停药后可自行缓解; TL会导致骨质疏松，对骨骼造成不可逆转的损害; 并有骨髓抑制作用，可引起白细胞、红细胞及血小板减少，严重者可发生粒细胞缺乏、贫血和再生障碍性贫血; 还可导致对生殖系统的损害，造成不孕不育。此外，还可能导致肝、心等器官功能衰竭，有时会发生皮肤变态反应、血栓性浅静脉炎等^[15-17]^。由于TL具有较大的毒副作用，临床的应用受到限制，降低毒副作用的同时提高疗效是急需解决的关键问题。现科研工作正在进一步加强对雷公藤的药物化学、药理作用及临床应用的研究，以期提取、纯化、合成出高效低毒的TL，提高其安全性和应用价值。

## 5 展望

中草药的使用在我国源远流长，积累了丰富的经验。深入研究植物提取物的抗肿瘤特性为筛选出新的安全有效的抗癌药提供了契机。TL的水溶性衍生物PG490-88正在进行治疗前列腺癌的临床试验^[18]^，具有广阔的应用前景。

随着现代化城市的不断发展，空气污染、吸烟等对人类的健康造成极大的危害，肺癌已经成为严重威胁人类健康的疾病之一。TL作为一种传统中药，在肺癌的治疗中具有巨大的潜力。尽管对TL抑制NSCLC的部分机制初有认识，但其作用机制尚未得到充分阐明，目前国内外在此方面研究不多，寄望日后深入研究。相信随着TL防癌、抗癌机制不断被深入研究和认可，TL有望成为NSCLC临床治疗中的新型药物。
